# The feasibility, acceptability, and usability of telehealth visits

**DOI:** 10.3389/fmed.2023.1198096

**Published:** 2023-07-19

**Authors:** Naina Sinha Gregory, Alpana P. Shukla, Jahi J. Noel, Laura C. Alonso, Jerad Moxley, Andrew J. Crawford, Peter Martin, Sonal Kumar, John P. Leonard, Sara J. Czaja

**Affiliations:** ^1^Division of Endocrinology, Diabetes and Metabolism, Weill Cornell Medicine, New York City, NY, United States; ^2^Division of Endocrinology, Diabetes and Metabolism, Comprehensive Weight Control Center, Weill Cornell Medicine, New York City, NY, United States; ^3^College of Agriculture and Life Science, Cornell University, Ithaca, NY, United States; ^4^Division of Geriatric and Palliative Medicine, Weill Cornell Medicine, New York City, NY, United States; ^5^Division of Hematology/Oncology, Weill Cornell Medicine, New York City, NY, United States; ^6^Division of Gastroenterology and Hepatology, Weill Cornell Medicine, New York City, NY, United States

**Keywords:** telehealth, telemedicine, virtual visits, video visits, COVID-19

## Abstract

**Background:**

Telemedicine is now common practice for many fields of medicine, but questions remain as to whether telemedicine will continue as an important patient care modality once COVID-19 becomes endemic. We explored provider and patients’ perspectives on telemedicine implementation.

**Methods:**

Physicians from three specialties within the Department of Medicine of a single institution were electronically surveyed regarding their perceptions of satisfaction, benefits, and challenges of video visits, as well as the quality of interactions with patients. Patients were surveyed via telephone by the Survey Research Group at Cornell about participation in video visits, challenges encountered, perceived benefits, preferences for care, and overall satisfaction.

**Results:**

Providers reported an overwhelmingly positive experience with video visits, with the vast majority agreeing that they were comfortable with the modality (98%) and that it was easy to interact with patients (92%). Most providers (72%) wanted to have more telemedicine encounters in the future. Key factors interfering with successful telemedicine encounters were technical challenges and insufficient technical support. Overall, patients also perceived video visits very positively regarding ease of communication and care received and had few privacy concerns. Some (10%–15%) patients expressed interest in receiving more technical support and training. There was a gradient of satisfaction with telemedicine across specialties with patients receiving weight management reporting more favorable responses while patients with lymphoma expressed more mixed responses.

**Conclusion:**

Both providers and patients found telemedicine to be an acceptable and useful modality to provide or receive medical care. The principal barrier to successful encounters was technical challenges.

## Introduction

The COVID-19 pandemic dramatically changed the administration of patient care as seen in the rising use of telemedicine and other online health services (1, 2). The virtual modality reduced face-to-face patient interactions to help mitigate the spread of the SARS-CoV-2 virus. Telemedicine is currently common practice for many fields of medicine, but questions remain as to whether telemedicine will continue as an important patient care modality once COVID-19 becomes endemic (3, 4). Important issues to consider include understanding barriers and facilitators to the continued successful implementation of telemedicine (5–7); and whether providers’ and patients’ attitudes support continued use of telemedicine, and in which contexts (3, 4, 8, 9).

Differences in medical care paradigms suggest that some specialties may be better suited to the use of telemedicine than others (1, 4, 8–11). For example, chiropractic therapy is less suited for telemedicine due to the hands-on nature of the practice, but psychiatric evaluations can be readily performed through a video visit. Even within some fields, certain visits are more amenable to telemedicine; for example, surgery must happen in person, but surgical follow up can be successfully achieved by a video visit. Some research studies have assessed providers’ attitudes toward telemedicine and perceptions of efficacy of telemedicine patient visits (2, 12, 13) or patients (2, 12, 13), but there are still existing gaps in knowledge in this area, including information regarding which types of visits are best suited to the video modality.

We performed a survey-based study from October 2020 to April 2021 of three internal medicine subspecialties (Endocrinology, Gastroenterology, and Hematology/Medical Oncology) at an academic medical center in New York to explore the perceptions of video visits and challenges to implementation. A unique aspect of our study is that we obtained the perspective of both providers and patients. We also collected an in-depth exploration of the specific challenges experienced during the use of telemedicine. Additionally, we assessed satisfaction with telemedicine visits for both the providers and the patients, gathering data across various types of visits and specialties. Taken together, our data inform future uses of telemedicine as well as areas of improvement to optimize the use of this modality for healthcare delivery.

## Methods

An anonymous Redcap survey was emailed to physicians from three specialties within the Department of Medicine at Weill Cornell Medicine: Gastroenterology, Endocrinology, and Hematology/Oncology. The survey contained 21 items related to perceptions of satisfaction with video/telehealth visits, benefits of video visits, challenges with video visits, and quality of interactions with patients. The responses for 13 of the items were rated on a 5-point Likert scale ranging from strongly agree to strongly disagree. Four of the items were open-ended including perceptions of benefits and challenges to video visits; patient populations experiencing challenges engaging in video visits; and types of patient interactions for which video visits were appropriate.

A patient survey was also administered to patients of the physicians. The patient survey was administered via telephone by the Survey Research Group at Cornell University. Patients were identified by the providers in each of the subspecialties. Initially they were sent a letter from their provider informing them about the study and that they were going to be contacted by the Survey Research Institute. The patients were contacted up to three times via telephone. All patient responses were de-identified. The patient survey contained 25 items that were related to participation in video visits with their provider and addressed challenges encountered during the visits, perceived benefits of the video visits, preferences for care interactions, and overall satisfaction with the video visits. Responses to the items were rated on a 5-point Likert scale ranging from strongly agree to strongly disagree.

### Analysis

Responses to the provider and patient survey data were initially summarized using descriptive statistics such as percentages, means and standard deviations (where appropriate, e.g., age). Group differences (e.g., provider specialty, patient age groups) were analyzed using a between subjects ANOVA. For these analyses we used the entire scale range from strongly agree to strongly disagree but for clarity of presentation, we collapsed these categories into agree, neither agree nor disagree, and disagree and presented % agree in the tables, with the exception of Table 1.

**Table 1:** Patient perceptions of video visits (*N* = 221).

	Agree	Neither agree nor disagree	Disagree
Easy to communicate	205 (93%)	4 (2%)	12 (5%)
Received care I need	195 (88%)	11 (5%)	14 (6%)
My questions were answered	206 (93%)	8 (4%)	7 (3%)
Had privacy concerns	19(8%)	4 (2%)	198 (90%)
Satisfied with the interaction	202 (92%)	6 (3%)	12 (6%)
Difficult to understand	12 (5%)	6 (3%)	203 (92%)
Difficult to hear	10 (4%)	4 (2%)	207 (94%)
Difficult to see	20 (9%)	2 (1%)	199 (90%)
Needed technical support	25 (11%)	12 (5%)	184 (83%)
Interested in more training	29 (14%)	15 (7%)	175 (79%)

The provider qualitative data for each of the four opened queries was analyzed by independent raters. Initially the data for each question were coded for themes. Then the two independent raters coded the data into the thematic categories. The inter-rater reliability was high (Κ = 0.7). Cases of disagreement among the raters was adjudicated by a third party.

## Results

### Results of provider survey

Fifty-one out of 79 providers (65%) responded from three different internal medicine subspecialities: gastroenterology (49%), endocrinology (42%) and hematology/oncology (9%). The response rate by division was 50% gastroenterology (20/40), 68% endocrinology (23/34) and 80% hematology (4/5). Four providers did not provide a specialty.

The majority of providers agreed that they were comfortable with the video visit modality (98%) and that it was easy to interact with patients (92%; see Table 2). More than 85% believed that they were receiving accurate information from patients and that the patients understood the information that was being conveyed. Most providers (72%) wanted to have more telemedicine encounters in the future and 88% found the overall experience positive. However only 67% of the providers felt confident that most patients were able to use the videoconferencing devices. Examination of the qualitative data regarding the benefits of video visits indicated that the 55% of the providers felt that cost savings and convenience were major benefits associated with video visits and 43% felt that video visits provided broader access to patients as well as enhanced the patient’s ability to access care. In addition, 25% of the providers indicated that video visits allowed for social distancing and kept patients safe (see Table 3). The following are examples of noted benefits gleaned from the qualitative data.

**Table 2:** Provider perceptions of telemedicine visits (*N* = 57).

	Gastro	Endo	Hema
	% agree (*N*)	% agree (*N*)	% agree(*N*)
I felt that videoconferencing made it easy for me to interact with my patients.^*^	96% (22)	90% (18)	75% (3)
I felt comfortable using videoconferencing to interact with my patients.^**^	100% (23)	95% (19)	100% (4)
I felt confident that I was receiving accurate information from my patients during the videoconferencing visits.^**^	100% (23)	75% (15)	50% (2)
I felt confident my patients understood what I was saying during the videoconference visits.^*^	100% (23)	80% (16)	75% (3)
I would like to have more telemedicine visits with my patients in the future.	74% (17)	70% (14)	50% (2)
Overall, my experience with telemedicine was positive.^**^	100% (23)	75% (15)	75% (3)
I found it difficult to communicate with my patients using videoconferencing visits.^**^	0% (0)	10% (2)	50% (2)
I frequently encountered technical challenges during my videoconferencing visits with my patients.^**^	43% (10)	85% (17)	75% (3)
I received sufficient technical support during my videoconferencing visits with my patients.	30% (7)	10% (2)	1 (25%)
I felt confident that the majority (~80%) of my patients/clients were able to use the videoconferencing devices.^*^	78% (18)	50% (10)	75% (3)
The limitations in laboratory testing resulted in a major limitation to the value of my video visits.	13% (3)	15% (3)	75% (3)
The limitations in conducting a physical examination resulted in a major limitation in the value of my video visits.	9% (2)	20% (4)	50% (2)
I think video visits take more time than in person visits.^**^	9% (2)	30% (6)	0% (0)
I anticipate that ____ % of my patient visits will be video visits in the future.	34% *(20%)*	*44% (17%)*	*1*6% *(%)*

*, Group differences statistically significant at *p* < 0.05. **, Group differences statistically significant at *p* < 0.01.

**Table 3:** Provider perceptions of benefits of video visit (*N* = 49).

	Total % (*N*)	GI %	Endo %	Hema %
Patient safety and social distancing	25% (12)	22% (5)	26% (5)	50% (2)
Broader access to patients/enhanced patient ability to access care	43% (21)	44% (10)	58% (11)	0 (0)
Cost savings and convenience (e.g., no need for patient to take off work; reduced need to travel; ease of scheduling)	55% (27)	74% (17)	37% (7)	50% (2)
Improved visit adherence	12% (6)	9% (2)	21% (4)	0 (0)
Facilitates ability to stay connected with patients	16% (8)	13% (3)	26% (5)	0 (0)

“*The video visits keep patients safe during the pandemic and still engaged in care, we can also reach patients from a further radius.”*

“*Video visits are less time consuming and expensive for patients.”*

*“Video visits allow patients to be seen in between yearly in person visits.”*

When exploring the issues perceived as challenges to successful video visits 80% of the providers indicated inadequate technical support and close to 2/3 indicated that they frequently encountered technical challenges (see Table 4). The most common types of technical challenges included the inefficient or challenging design of the video visit system (49%), poor internet connectivity (32%) and poor audio/visual quality (30%). The following quotes from responses to the qualitative questions demonstrate examples of perceptions of challenges associated with the video visits.

**Table 4:** Types of technical challenges (*N* = 37).

	Total % (*N*)	GI %	Endo %	Hema %
Patient lack of access to needed technology	19% (7)	13% (2)	25% (4)	0% (0)
Poor internet connectivity	32% (12)	19% (3)	50% (8)	0% (0)
Poor audio/visual quality	30% (11)	31% (5)	31% (5)	33% (1)
Provider had technical problems	16% (3)	13% (2)	0.06% (1)	0% (0)
Inefficient or challenging system design (e.g., patient unable to log in or connect audio)	49% (18)	42% (7)	56% (9)	67% (2)

*“Problems with Internet and connectivity issues at both my end and the patient end.”*

*“Tech support was hard to come by. I had little understanding of what it looked like from patients’ side and no way to help them.”*

*“It was difficult for patients to log in and there was no way to message the patient in real time. We need a text messaging solution.”*

With respect to provider perceived types of patients for whom video visits were most challenging, 86% of the providers indicated older patients and 37% indicated patients that lacked technology skills or internet/system access. Eighteen percent of the providers found that limitations in lab testing and the ability to perform a physical exam constituted a major limitation to the value of video visits. For example, one of the providers indicated that “labs and physical exams are needed for sicker patients and that is a limitation to telehealth visits.” A similar percentage felt that video visits took more time than an in-person visit. Despite the perceived limitations providers estimated that 44% (SD = 19%, Range = 10%–85%) of their future patient visits would be video visits.

When the responses from the individual specialties were examined, there were some significant differences between gastroenterology and endocrinology. Hematology/oncology was not included in the subgroup analysis due to a low number of respondents. Gastroenterologists felt more comfortable using videoconferencing to interact with patients [*t*(41) = 3.2, *p* = 0.003], more confident that they were receiving accurate information from patients [*t*(41) = 4.0, *p* < 0.001], and had an overall more positive experience with telemedicine than their endocrinology counterparts [*t*(41) = 3.4, *p* = 0.002]. Gastroenterologists felt that video conferencing made it easy to interact with patients [*t*(41) = 2.0, *p* = 0.046], and felt confident that patients understood what was being said [*t*(41) = 2.2, *p* = 0.03; Table 2].

Although both divisions had overall positive responses to video visits, significantly more endocrinologists encountered technical challenges [*t*(41) = −3.2, *p* = 0.002] and found it difficult to communicate with patients using videoconferencing [*t*(41) = −3.3, *p* = 0.002]. Endocrinologists also thought video visits took more time than in-person visits [*t*(41) = 2.8, *p* = 0.009].

When the gastroenterologists were queried about the type of patients that would be good candidates for telemedicine visits 92% thought they could perform adequate health maintenance for IBD and chronic liver patients, 86% thought they would be able to monitor for worsening or progressive disease and 67% felt they could identify patients who needed to schedule infusions and improve adherence to scheduled therapy.

Some of the endocrinologists identified the type of patient interactions most suitable for telehealth with the following responses.

“*Stable chronic disease like hypothyroidism, osteoporosis and diabetes can be used for check-ins in between yearly or six-month follow-ups for more frequent patients who need dose titrations of insulin.”*

*“It is ideal for follow-up of endocrine conditions that require a lot of data interpretation and discussions with patients. Perfect for osteoporosis and diabetes follow-up patients. Great option for second opinions. Overall, it is very helpful.”*

### Results of patient survey

A total of 240 patients including 68% white, 12% African American, 9% Hispanic, and 6% that identified as mixed race or other were surveyed. The sample ranged in age from 19 to 94 years (*M =* 57.5, *SD = 17.4*). Forty-two percent of the sample were 65 years of age or older. The majority (63%) of the sample was female and fairly well-educated, 78% had a college degree or beyond.

Given that the providers indicated that older patients experienced the most challenges with video visits, we categorized the sample into three age groups (<46, 46–64, and >65 years) to examine potential age differences in technology experience. Thirty percent of the sample was <46 years. of age, 28% were between the ages of 46–64, and 42% were aged 65 and older. Overall, approximately two-thirds had experience using all modalities of technology including computer, mobile devices, and the internet. Patients in the older age group reported that they had significantly less computer experience [*F*(2, 226) = 6.5, *p* < 0.002] and mobile device experience [*F*(2,226) = 7.2, *p* < 0.001] than the other age groups. Not surprisingly, patients under the age of 45 reported significantly more internet experience than patients in the other age groups [*F*(2,226) = 3.9, *p* < 0.02].

Of the 240 patients surveyed, 90% had participated in video visits. The remaining 10% may have had either audio calls or failed tele-visits. Lack of internet access and preference for face-to-face visits were main reasons cited for not participating in a video visit. Overall, the patients perceived video visits very positively and indicated that it was easy to communicate with their providers, were satisfied with the care received, and most had no privacy concerns (see Table 1). Approximately 10%–15% of patients expressed interest in receiving more technical support and training. There was difference in the patient’s age among the 3 specialties [*F*(2,232) = 6.8, *p* = 0.001]. The endocrine specialty had younger patients than either gastroenterology (*p* = 0.001) or lymphoma (*p* = 0.002).

Patients from the different specialties were asked their views on the role of video visits in their medical care. Approximately 76%–88% of gastroenterology patients felt that telemedicine was good for follow-up visits, discussing lab results and monitoring, but only 46% thought that it was sufficient to accomplish appointment goals and needs. The majority of the endocrine patients found video visits to be a good way to discuss blood glucose control, lab results, and thyroid, osteoporosis, and other endocrine disorders. Over 75% of endocrine patients were satisfied that their diabetes related questions were answered during their video visits and felt that this option would help them to better maintain more regular follow-ups. Twenty-two percent of the endocrine patients, however, did not perceive that telemedicine is a good modality to start new medication. The majority (85%) of weight management patients endorsed the utility of video visits for lifestyle counseling (including diet and exercise), discussing weight management, the adjustment of medications, and answering questions related to weight management. Approximately 50% of hematology patients agreed that a video visit was good for monitoring treatment and for having a follow-up after completion of treatment but only 25% felt that it was sufficient to accomplish their appointment treatment goals.

## Discussion

This study assessed perceptions of telemedicine visits from both provider and patient perspectives in three internal medicine subspecialities. The results show that providers and patients that participated in videoconferencing health services had an overall positive experience. Providers and patients rated video visits very highly for their ease of communication, and ability to fulfill patient needs and appointment goals especially for health management tasks such as reviewing labs, monitoring, and follow-up visits. These types of activities appear to be the most suited for via telemedicine visits and the ones that will contribute to the sustainability of telehealth services. Overall, most patients reported no concerns related to privacy. Furthermore, the most providers surveyed indicated that they would like telemedicine integrated into their practice in the future, despite the return of safe in person, face to face care options.

Optimal application of the telemedicine modality may depend on factors intrinsic to the patient and their current needs. These results agree with previous studies investigating the feasibility and acceptability of telemedicine across subspecialities (1, 2, 4, 7, 8, 10, 12–16). Previous studies have investigated the variability of acceptability of telemedicine between subspecialties of medicine with one reporting that 84.4% (*n* = 129) and 82.9% (*n* = 94) of internal medicine patients and provider, respectively, said they enjoyed tele-visits as compared to 94% (*n* = 94) and 64% (*n* = 25) of patients and providers, respectively, in family medicine (13). An analysis of telehealth satisfaction among patients undergoing cancer rehabilitation found that 94.8% (*n* = 76) agreed (with responses such as “quite a bit” or “very much”) that tele-visits were a positive experience as did 63.1% (*n* = 155) of providers. About 84% of the of providers reported that the main patient issues of the visit were adequately addressed during the visit (12). On the other hand, patients undergoing cancer diagnosis and treatment expressed that they did not want to be told bad news over a video/phone visit, despite having an otherwise positive response to telehealth (15). Additionally, a study examining clinician satisfaction found that at least 25% (*n* = 112) of clinicians would like to see tele-visits implemented into their practice in the future (14).

We observed a gradient of patient satisfaction among the internal medicine subspecialities, with weight management patients indicating more positive responses while lymphoma patients relaying more mixed experiences with telehealth services. Our findings are consistent with other centers that have reported high levels of patient satisfaction with the use of telemedicine for weight management (17, 18). GI providers expressed greater satisfaction than Endocrine providers. During the study period GI providers were still offering in-person visits for those whose conditions/treatments deemed the service necessary for in person care. In contrast, Endocrine was completely online at this time. These signals suggest that the severity and acuity of the disease, and the reason for the appointment should be taken into consideration when offering telehealth services. Additionally, these results favor a hybrid treatment model.

Our study found that tele-visits are a generally positive experience for both providers and patients. However, importantly the findings also indicated challenges associated with telehealth. The higher rate of these issues may have been intrinsic to the initial platform. In September 2020, the zoom platform was adopted and throughout the course of this study the platform was continually optimized and refined. The introduction of the technology in a rapid manner due to urgency associated with COVID-19 and the switching between platforms may have contributed to confusion and technical challenges. However, it should be noted that the providers reported that the older adult patients encountered the most challenges and as noted these patients had significantly less technology experience. Technology issues, such as lack of internet access or poor-quality video/audio (7), were the most prominent challenges for both providers and patients. This is in-line with the findings of Chang and colleagues (12) who found that a common patient barrier to use of telehealth, especially among underserved populations was lack of reliable internet as well as comfort with technology. Our findings also indicated that patients with low technical skills also experienced challenges with the telehealth visits. The providers in our sample also indicated that the video visits were particularly challenging for older patients. The older patients in our sample had significantly lower technology experience than the younger and middle-aged patients. These findings are consistent with recent data from the Pew Research Center (19) that although rates of technology adoption are increasing among older adults an age-related digit divide still exists (7). Given the increased reliance on telehealth for health management activities and that older adults tend to require more health care services, strategies need to be in place to ensure that people in all age groups have “meaningful” access to telehealth technology.

Successful implementation of telehealth for all patient populations requires telehealth systems to be designed for ease of use for patients with low technology skills and low technology efficacy. Success also requires that patients have access to the internet, appropriate technology, adequate training, and technical support. Unaddressed patient challenges with telehealth may exacerbate health inequities. Healthcare providers must also have access to easy-to-use systems, training, and technical support. In essence, telehealth systems should be designed using a user-centered design approach that involves participation in the design process from all user groups including both patients and providers (5).

This study delineates both provider and patient perspectives regarding feasibility, acceptability, and utility of telemedicine across three medical specialties. The findings also provide information for the types of healthcare tasks for which telehealth visits are most suited and the patient populations for whom these visits are challenging. In addition, the findings suggest common types of challenges associated with telehealth. The study also has some limitations. The sample was primarily white with high levels of technology experience. It was also restricted to a few specialties at one academic medical center. Patient data on participation in visits was based on self-report. Further, this study was performed over the course of one time period, so it is not possible to examine how perceptions of telehealth changed over time as both providers and patients had more experience with tele-visits and perhaps the technology being used has improved. This would be an interesting follow-up study. Despite these weaknesses, the findings expand our understanding of the benefits and challenges associated with telehealth from both the patient and provider prospective. The COVID-19 pandemic sparked a rapid adoption of telehealth services. As we adjust to a new post pandemic normal, our findings suggest that telehealth will most likely remain an important and valued modality for ambulatory patient care.

## Data availability statement

The original contributions presented in the study are included in the article/supplementary material, further inquiries can be directed to the corresponding author.

## Ethics statement

The studies involving human participants were reviewed and approved by Weill Cornell IRB. The patients/participants provided their written informed consent to participate in this study.

## Author contributions

All authors listed have made a substantial, direct, and intellectual contribution to the work and approved it for publication.

## Conflict of interest

The authors declare that the research was conducted in the absence of any commercial or financial relationships that could be construed as a potential conflict of interest.

## Publisher’s note

All claims expressed in this article are solely those of the authors and do not necessarily represent those of their affiliated organizations, or those of the publisher, the editors and the reviewers. Any product that may be evaluated in this article, or claim that may be made by its manufacturer, is not guaranteed or endorsed by the publisher.

## References

[1] Bitar H Alismail S. The role of eHealth, telehealth, and telemedicine for chronic disease patients during COVID-19 pandemic: a rapid systematic review. Digit Health. (2021) 7:205520762110093. doi: 10.1177/20552076211009396, PMID: 33959378 PMC8060773

[2] Garfan S Alamoodi AH Zaidan BB Al-Zobbi M Hamid RA Alwan JK. Telehealth utilization during the Covid-19 pandemic: a systematic review. Comput Biol Med. (2021) 138:104878. doi: 10.1016/j.compbiomed.2021.104878 34592585 PMC8450049

[3] Seifert A Batsis JA Smith AC. Telemedicine in long-term care facilities during and beyond COVID-19: challenges caused by the Digital divide. Front Public Health. (2020) 8:1595. doi: 10.3389/fpubh.2020.601595 PMC7649197 33194999

[4] Shirke MM Shaikh SA Harky A. Implications of telemedicine in oncology during the COVID-19 pandemic. Acta Biomed. (2020) 91:e2020022. doi: 10.23750/abm.v91i3.9849 PMC7716949 32921719

[5] Anthony DL Campos-Castillo C Lim PS. Who Isn’t using patient portals and why? Evidence and implications from a National Sample of US adults. Health Aff. (2018) 37:1948–54. doi: 10.1377/hlthaff.2018.05117, PMID: 30633673

[6] Şahin E Yavuz Veizi BG Naharci MI. Telemedicine interventions for older adults: a systematic review. J Telemed Telecare. (2021):1357633X2110583. doi: 10.1177/1357633X211058340 34825609

[7] Vogels E.A. Perrin A. Rainie L. Anderson M.. 53% of Americans say the internet has been essential during the COVID-19 outbreak. Pew Research Center: Internet, Science & Tech. (2020). Available at: https://www.pewresearch.org/internet/2020/04/30/53-of-americans-say-the-internet-has-been-essential-during-the-covid-19-outbreak/.

[8] Bohingamu Mudiyanselage S Stevens J Watts JJ Toscano J Kotowicz MA Steinfort CL. Personalised telehealth intervention for chronic disease management: a pilot randomised controlled trial. J Telemed Telecare. (2018) 25:343–52. doi: 10.1177/1357633X18775850, PMID: 29793387

[9] Wootton R. Twenty years of telemedicine in chronic disease management–an evidence synthesis. J Telemed Telecare. (2012) 18:211–20. doi: 10.1258/jtt.2012.120219, PMID: 22674020 PMC3366107

[10] Boscari F Ferretto S Uliana A Avogaro A Bruttomesso D. Efficacy of telemedicine for persons with type 1 diabetes during Covid19 lockdown. Nutr Diabetes. (2021) 11. doi: 10.1038/s41387-020-00147-8 PMC7790327 33414391

[11] Sood A Watts SA Johnson JK Hirth S Aron DC. Telemedicine consultation for patients with diabetes mellitus: a cluster randomised controlled trial. J Telemed Telecare. (2017) 24:385–91. doi: 10.1177/1357633X17704346 28406066

[12] Chang PJ Jay GM Kalpakjian C Andrews C Smith S. Patient and provider-reported satisfaction of Cancer rehabilitation telemedicine visits during the COVID -19 pandemic. PMR. (2021) 13:1362–8. doi: 10.1002/pmrj.12552, PMID: 33455066 PMC8013293

[13] Volcy J Smith W Mills K Peterson A Kene-Ewulu I McNair M. Assessment of patient and provider satisfaction with the change to telehealth from in-person visits at an academic safety net institution during the COVID-19 pandemic. J Am Board Fam Med. (2021) 34:S71–6. doi: 10.3122/jabfm.2021.S1.200393, PMID: 33622821

[14] Gentry MT Puspitasari AJ McKean AJ Williams MD Breitinger S Geske JR. Clinician satisfaction with rapid adoption and implementation of telehealth services during the COVID-19 pandemic. Telemed e-Health. (2021) 27:1385–92. doi: 10.1089/tmj.2020.0575 33606560

[15] Smrke A Younger E Wilson R Husson O Farag S Merry E. Telemedicine during the COVID-19 pandemic: impact on Care for Rare Cancers. JCO Glob Oncol. (2020) 6:1046–51. doi: 10.1200/go.20.00220, PMID: 32639877 PMC7392777

[16] Eberly LA Kallan MJ Julien HM Haynes N Khatana SAM Nathan AS. Patient Characteristics Associated With Telemedicine Access for Primary and Specialty Ambulatory Care During the COVID-19 Pandemic. JAMA Netw Open. (2020) 3:e2031640. doi: 10.1001/jamanetworkopen.2020.31640, PMID: 33372974 PMC7772717

[17] Lohnberg JA Salcido L Frayne S Mahtani N Bates C Hauser ME. Rapid conversion to virtual obesity care in COVID-19: impact on patient care, interdisciplinary collaboration, and training. Obes Sci Pract. (2021) 8:131–6. doi: 10.1002/osp4.550, PMID: 34540265 PMC8441727

[18] Vosburg RW Robinson KA Gao C Kim JJ. Patient and provider satisfaction with telemedicine in a comprehensive weight management program. Telemed J e-Health. (2022) 28:384–90. doi: 10.1089/tmj.2021.0077 33913743

[19] Faverio M.. Share of those 65 and older who are tech users has grown in the past decade. Pew Research Center. (2022). Available at: https://www.pewresearch.org/fact-tank/2022/01/13/share-of-those-65-and-older-who-are-tech-users-has-grown-in-the-past-decade/.

